# Zika virus infection of cellular components of the blood-retinal barriers: implications for viral associated congenital ocular disease

**DOI:** 10.1186/s12974-017-0824-7

**Published:** 2017-03-03

**Authors:** Tracoyia Roach, Donald J. Alcendor

**Affiliations:** Department of Microbiology and Immunology, Center for AIDS Health Disparities Research, School of Medicine, Meharry Medical College, 1005 Dr. D.B. Todd Jr. Blvd., Nashville, TN 37208-3599 USA

**Keywords:** Zika virus, Blood-retinal barrier, Retinal endothelial cells, Retinal pigmented epithelial cells, Retinal pericytes, Cytokines, Inflammation

## Abstract

**Background:**

Ocular abnormalities present in microcephalic infants with presumed Zika virus (ZIKV) congenital disease includes focal pigment mottling of the retina, chorioretinal atrophy, optic nerve abnormalities, and lens dislocation. Target cells in the ocular compartment for ZIKV infectivity are unknown. The cellular response of ocular cells to ZIKV infection has not been described. Mechanisms for viral dissemination in the ocular compartment of ZIKV-infected infants and adults have not been reported. Here, we identify target cells for ZIKV infectivity in both the inner and outer blood-retinal barriers (IBRB and OBRB), describe the cytokine expression profile in the IBRB after ZIKV exposure, and propose a mechanism for viral dissemination in the retina.

**Methods:**

We expose primary cellular components of the IBRB including human retinal microvascular endothelial cells, retinal pericytes, and Müller cells as well as retinal pigmented epithelial cells of the OBRB to the PRVABC56 strain of ZIKV. Viral infectivity was analyzed by microscopy, immunofluorescence, and reverse transcription polymerase chain reaction (RT-PCR and qRT-PCR). Angiogenic and proinflammatory cytokines were measured by Luminex assays.

**Results:**

We find by immunofluorescent staining using the Flavivirus 4G2 monoclonal antibody that retinal endothelial cells and pericytes of the IBRB and retinal pigmented epithelial cells of the OBRB are fully permissive for ZIKV infection but not Müller cells when compared to mock-infected controls. We confirmed ZIKV infectivity in retinal endothelial cells, retinal pericytes, and retinal pigmented epithelial cells by RT-PCR and qRT-PCR using ZIKV-specific oligonucleotide primers. Expression profiles by Luminex assays in retinal endothelial cells infected with ZIKV revealed a marginal increase in levels of beta-2 microglobulin (β2-m), granulocyte macrophage colony-stimulating factor (GMCSF), intercellular adhesion molecule 1 (ICAM-1), interleukin-6 (IL-6), monocyte chemotactic protein-1 (MCP1), and vascular cell adhesion molecule 1 (VCAM-1) and higher levels of regulated upon activation, normal T cell expressed and presumably secreted (RANTES) but lower levels of interleukin-4 (IL-4) compared to controls.

**Conclusions:**

Retinal endothelial cells, retinal pericytes, and retinal pigmented epithelial cells are fully permissive for ZIKV lytic replication and are primary target cells in the retinal barriers for infection. ZIKV infection of retinal endothelial cells and retinal pericytes induces significantly higher levels of RANTES that likely contributes to ocular inflammation.

## Background

Zika virus (ZIKV) is an arbovirus that belongs to the Flavivirus family which also includes West Nile virus, dengue, yellow fever, and Japanese encephalitis viruses and is transmitted to humans by Aedes species mosquitoes [1, 2]. ZIKV was first identified in a rhesus monkey in 1947 and first recognized in humans in 1952 [1, 3]. ZIKV has quickly spread to more than 70 countries in the Americas and the Caribbean infecting more than 2 million people [4, 5]. Currently, there is no treatment or vaccine for Zika virus. There is very limited information known about this emerging global health threat.

ZIKV infection has been associated with a sporadic increase in the incidence of microcephaly in infants [6–9]. Congenital ocular findings concomitant with microcephaly have also been associated with ZIKV infection during pregnancy [10–13]. A recent study showed ocular abnormalities present in 34.5% of microcephalic infants examined and involved the bilateral vision in 70% of them [10]. The lesions included focal pigment mottling of the retina, chorioretinal atrophy, optic nerve abnormalities, bilateral iris coloboma (congenital fissure), and lens dislocation [10]. These lesions are considered vision-threatening, and children should be screened as a process of differential diagnosis to rule out other causes such as West Nile virus infection, toxoplasmosis, cytomegalovirus, rubella, herpes simplex virus, and syphilis [13, 14]. Children born to mothers with little or no symptoms of ZIKV infection can still have microcephalic babies with severe ocular abnormalities [15]. This finding would support the notion of ophthalmic screening for all babies born in epidemic regions. Risk factors for ocular involvement in infants with presumed ZIKV congenital infection include smaller cephalic diameters at birth and infants whose mother develop symptoms during the first trimester of pregnancy [16]. Adults with acute ZIKV disease often experience hyperemic sclera, conjunctivitis, and retro-orbital pain, and uveitis has also been observed in a patient with ZIKV infection after an initial clinical presentation of conjunctival hyperemia [17–19]. The target cells for ZIKV-associated ocular disease are unknown. The cytokine dysregulation that contributes to ZIKV-induced ocular inflammation is yet to be identified. The route of viral dissemination in the ocular compartment has not been described. Here, we identify target cells in both the inner and outer blood-retinal barriers (IBRB and OBRB), describe the cytokine expression profile in retinal endothelial cells after ZIKV exposure, and propose a mechanism for viral dissemination in the retina.

## Methods

### Cells

Human primary retinal microvascular endothelial cells and retinal pericytes were obtained from Cell Systems Corporation (Kirkland, WA, USA) and were cultivated in Pericyte Media (PM) from ScienCell (Carlsbad, CA, USA). Primary human retinal pigmented epithelial cells and epithelial cell media (EpiCM) were obtained from ScienCell. The human Müller cell line MIO-M1 [20], derived from an adult retina, was kindly provided by Dr. John Penn (Vanderbilt University Medical Center Eye Institute). Acquisition of the MIO-M1 cell line was approved by the Internal Review Board and Ethics Committee of Vanderbilt University Medical Center in Nashville, Tennessee. Retinal pericytes and retinal endothelial cells were maintained at passage level 3 in PM media. The Müller cell line MIO-M1 was maintained in Dulbecco’s Modified Eagle Medium (DMEM) supplemented with 10% fetal bovine serum, 1% Pen/Strep. All cells were trypsinized and plated in uncoated 100-cm^2^ dishes or uncoated 4.2-cm^2^ glass chamber slides at a density of and 2.5 × 10^5^ cells per dish and well, respectively.

### Viruses and virus cultivation

The Zika virus strain PRVABC59 provided by the Centers for Disease Control and Prevention (CDC) used in this study was originally isolated from a human serum specimen from Puerto Rico in December 2015, nucleotide (GenBank):KU501215 ZIKV strain PRVABC59, complete genome [21–23]. The virus was cultivated in Vero cells (*Cercopithecus aethiops*, African green monkey kidney cell line), and infectious supernatant was filtered using a 0.22-μm filter and the serum content adjusted to 15%. Viral titers were done by end-point dilution and infectivity measured by IFA staining with the 4G2 antibody (fluorescent focus assay (FFA) on Vero cells. Stock viral titer was adjusted to ~1 × 10^6^ FFU/5 μl of infectious culture supernatant. Heat-killed ZIKV was prepared by heating the viral inoculum at 65 °C for 30 min in a water bath [24]. The mild heat inactivation that we employ is unlikely to cause a global effect on thermolabile viral proteins. All experiments were carried out under biosafety level 2 containment as recommended. The use of ZIKV was approved by the Meharry Medical College Institutional Biosafety Committee.

### Antibody validation

ZIKV-infected Vero cells were used to validate the monoclonal antibody to the Flavivirus group antigen which binds to the fusion loop at the extremity of domain II of the E protein (D1-4G2-4-15, 4G2) (Millipore, Temecula, CA, USA) [25, 26]. ZIKV cytopathology in Vero cells included cell rounding and sloughing with a perinuclear staining profile using the 4G2 antibody by immunofluorescent staining (data not shown).

### Immunofluorescence

Immunofluorescent staining was performed as previously described [27]. Briefly, chamber slide cultures containing ZIKV-infected or mock-infected retinal endothelial cells, retinal pericytes, Müller cells, or retinal pigmented epithelial cells were washed twice with PBS pH 7.4, air-dried, and fixed in absolute methanol for 10 min. Cells were air-dried for 15 min, hydrated in Tris-buffered saline (pH 7.4) for 5 min, and incubated separately for 1 h with monoclonal antibodies to von Willebrand factor (VWF) for retinal endothelial cells (Millipore, Temecula, CA, USA), or vimentin for retinal pigmented epithelial cells (Santa Cruz, CA, USA). All antibodies were diluted 1:50 in PBS pH 7.4. For ZIKV infection of retinal endothelial cells, retinal pericytes, Müller cells, and retinal pigmented epithelial cells, cells were incubated for 1 h with monoclonal antibodies to the 4G2 Flavivirus group antigen, at a 1:50 dilution in PBS pH 7.4. Donkey anti-mouse secondary antibodies conjugated to fluorescein isothiocyanate (FITC) were used to detect ZIKV-positive cells. Immunofluorescent staining was performed as previously described [27].

### RT-PCR

Total RNA was extracted from both ZIKV-infected retinal endothelial cells and retinal pigmented epithelial cells along with their respective mock-infected and heat-killed ZIKV control cells using a Qiagen RNeasy Mini Kit (Qiagen, Valencia, CA, USA). RNA was DNase-treated prior to elution on the column according to the manufacturer’s recommendations. Messenger RNA in 0.5 μg of each sample was primed using oligo-dT and reverse-transcribed with a high-capacity complementary DNA (cDNA) reverse transcription kit (Applied Biosystems, Foster City, CA, USA). Gene-specific primer pairs included ZIKV forward primer 5′TTYGAAGCCCTTGGATTCTT3′ and ZIKV reverse primer 5′CYCGGCCAATCAGTTCATC3′ and 50 ng of cDNA for RT-PCR amplification, using PuReTaq Ready-To-Go PCR beads (GE Healthcare, Buckinghamshire, UK). PCR was carried out in a MJ Mini thermal cycler (Bio-Rad Laboratories, Hercules, CA, USA) in a final volume of 25 μl. The cycling protocol used was 95 °C for 5 min, 55 °C for 30 s, and 72 °C for 1 min for 36 cycles, with a final extension at 72 °C for 10 min. PCR products were electrophoresed in 1.5% agarose and DNA bands visualized by ethidium bromide. Primers for glyceraldehyde 3-phosphate dehydrogenase (GAPDH) forward primer 5′-TGATGACATCAAGAAGGTGGTGAA-3′ and reverse primer 5′-TCCTTGGAGGCCATGTGGGC CAT-3′ (256 bp) were amplified in mock and infected cells as a loading and quality control. Using ZIKV-infected cell total RNA, we amplified a 364-bp DNA fragment with the above primers, respectively, to positions 1538-1558 and 1902-1883 of the ZIKV genome sequence AY632535 [28].

### qRT-PCR

Total RNA was extracted separately from ZIKV-infected retinal endothelial cells, retinal pericytes, and Müller cells, along with the respective mock-infected controls using a Qiagen RNeasy Mini Kit (Qiagen, Valencia, CA, USA) as previously described above. Messenger RNA in 0.5 μg of each sample was primed using oligo-dT and reverse-transcribed with a high-capacity cDNA reverse transcription kit (Applied Biosystems, Foster City, CA, USA). Real-time quantitative PCR was performed on iCycler using iQ Sybr Green Supermix (Bio-Rad). Samples were analyzed in triplicate and normalized to GAPDH RNA. Reaction mixture contained 250 nM of each primer and 200 to 400 ng of template cDNA in a final volume of 20 μl. The primers specific for ZIKV were as follows: forward 5′-AGGATCATAGGTGATGAAGAAAAGT-3′ and reverse 5′-CCTGACAACACTAAGATTGGTGC-3′ [28]. RANTES primers used for qRT-PCR were as follows: forward 5′-GGCAGCCCTCGCTGTCATCCTCA-3′, reverse 5′-CTTGATGTGGGCACGGGGCAGTG-3′. GAPDH primers used for qRT-PCR were as follows: forward 5′-GAAGGTGAAGGTCGGAGT-3′ and reverse 5′-GAAGATGGTGATGGGATTTC-3′.

### Luminex assays

The inflammatory and angiogenic cytokine analysis was performed with 200 μl of supernatant from three pooled cultures of mock-infected, ZIKV-infected, and heat-killed ZIKV-exposed retinal endothelial cells for 96 h post-exposure using a Luminex instrument (Luminex Corporation, Austin, TX) and 100-plate viewer software. Luminex analysis on 47 different proinflammatory and angiogenic cytokines was performed on supernatants as previously described [29]. Infections were performed in triplicate in chamber slides for 96 h. Replicate assays are inherent in the Luminex technology by counting 50 bead replicates per analyte and reporting the median. This is the equivalent of running 50 replicate assays per well. In addition, robotic pipetting was performed for all volume-critical steps, which minimizes well-to-well variability, and calibrators and controls were run in duplicate involving three levels of control per analyte in duplicate on every plate [30]. Experiments presented in this study that involved ZIKV infections were performed in triplicate. Supernatants from mock-infected, ZIKV-infected, and heat-killed ZIKV-exposed retinal endothelial cells were separately taken from triplicate samples and pooled for Luminex analysis.

### Statistical analysis

Experiments presented in this study were performed in triplicate (mock-infected, ZIKV-infected, and heat-killed ZIKV-exposed retinal endothelial cells, retinal pericytes, Müller cells, and retinal pigmented epithelial cells were used for RT-PCR and qRT-PCR amplification of ZIKV and RANTES cDNA). To compare the mean values between the two groups, the unpaired *t* test was used. Statistical significance was defined as *P* < 0.05. Data are presented as means ± SD. qRT-PCR experiments were replicated three times and normalized to glyceraldehyde 3-phosphate dehydrogenase (GAPDH).

## Results

### Retinal endothelial and retinal pericytes are permissive for ZIKV infectivity but not retinal Müller cells

**Fig. 1:**
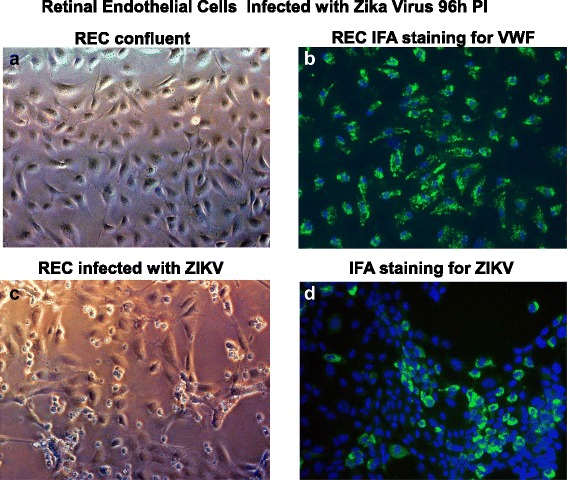
ZIKV infectivity of primary human retinal endothelial cells. Phase contrast images of **a** an uninfected confluent monolayer of retinal endothelial cells, **b** a confluent monolayer of retinal endothelial cells staining positive for von Willebrand factor (VWF), and **c** retinal endothelial cells 96 h after infection with the ZIKV. Immunofluorescence staining of ZIKV-infected endothelial cells after 96 h with the Flavivirus 4G2 antibody. **d** All images were taken on a Nikon TE2000S microscope mounted with a charge-coupled device (CCD) camera at ×200 magnification. For fluorescent images, 4′,6-diamidino-2-phenylindole (DAPI) was used to stain the nuclei blue

To identify target cells for ZIKV infection in the eye, we first examined cellular components of the retinal vascular unit that represents the IBRB and is a gateway to the retina. We exposed primary human retinal microvascular endothelial cells, retinal pericytes, and Müller cells to ZIKV at a multiplicity of infection (MOI) of 0.1 for 96 h. In mock-infected controls, we observed the normal cobblestone morphology of the retinal endothelial cells in confluent monolayer cultures (Fig. 1a). Retinal endothelial cells stained positive for the endothelial cell biomarker von Willebrand factor with strong staining of Weibel-Palade bodies (Fig. 1b). In retinal endothelial cells exposed to ZIKV, we observed cytopathic effects that included rounding and sloughing of cells with patches of the monolayer floating in the media (Fig. 1c). We confirmed ZIKA infectivity for retinal endothelial cells by immunofluorescent staining using the 4G2 monoclonal antibody in 96 h after infection (Fig. 1d). Virus-infected retinal endothelial cells showed a perinuclear staining profile with the 4G2 antibody (Fig. 1d). The 4G2 antibody was validated for reactivity in ZIKV-infected Vero cells (data not shown).

**Fig. 2:**
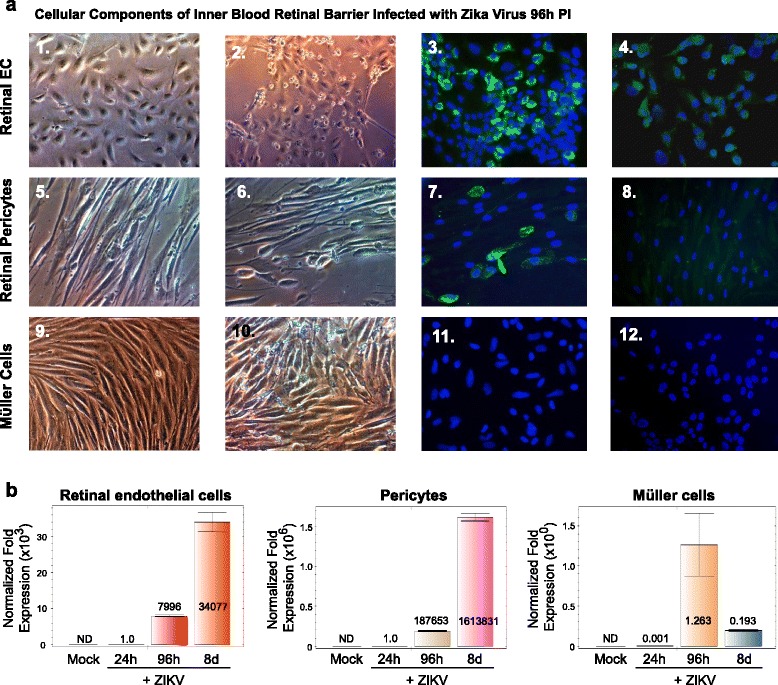
Cellular components of the inner blood-retinal barrier and ZIKV infectivity. Phase contrast images of **a** an uninfected confluent monolayer of retinal endothelial cells (**a-1**), a confluent monolayer of retinal endothelial cells infected with ZIKV 96 h after infection (**a-2**), immunofluorescence staining of ZIKV-infected endothelial cells with the Flavivirus 4G2 antibody (**a-3**), an uninfected confluent monolayer of retinal pericytes (**a-4**), a confluent monolayer of retinal pericytes infected with ZIKV 96 h after infection (**a-5**), immunofluorescence staining of ZIKV-infected pericytes with the Flavivirus 4G2 antibody (**a-6**), an uninfected confluent monolayer of Müller cells (**a-7**), a confluent monolayer of Müller cells infected with ZIKV 96 h after infection (**a-8**), and an immunofluorescence staining of ZIKV-infected Müller cells with the Flavivirus 4G2 antibody (**a-9**). Mock-infected controls of retinal endothelial cells (**a-4**), retinal pericytes (**a-8**), and Müller cells (**a-12**) stained with the 4G2 antibody. All images were taken on a Nikon TE2000S microscope mounted with a charge-coupled device (CCD) camera at ×200 total magnification. For fluorescent images, 4′,6-diamidino-2-phenylindole (DAPI) was used to stain the nuclei blue. **b** qRT-PCR time course of retinal endothelial cells, retinal pericytes, and Müller cells infected with ZIKV for 24 and 96 h and 8 days after infection. Mock-infected controls are also shown. All values were normalized to GAPDH. *ND* indicates no transcriptional expression detected

**Fig. 3:**
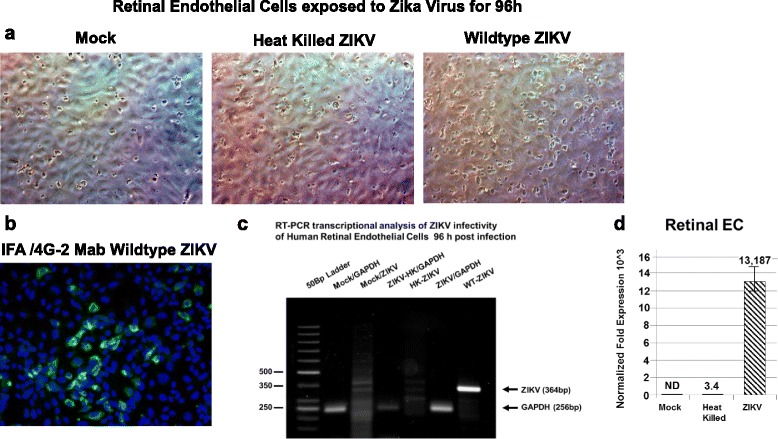
Retinal endothelial cells infectivity for ZIKV confirmed by RT-PCR. Phase contrast images of **a** a mock-infected confluent monolayer of retinal endothelial cells, a confluent monolayer of retinal endothelial cells exposed to heat-killed ZIKV, and retinal endothelial cells exposed to wild-type ZIKV. **b** Immunofluorescence staining of ZIKV-infected endothelial cells with the Flavivirus 4G2 antibody. **c** Semiquantitative RT-PCR amplification of a 364-bp fragment using ZIKV-specific primers. GAPDH was amplified as a control represented as a 256-bp fragment. Phase and fluorescent images were taken on a Nikon TE2000S microscope mounted with a charge-coupled device (CCD) camera at ×200 magnification. For fluorescent images, 4′,6-diamidino-2-phenylindole (DAPI) was used to stain the nuclei blue. **d** qRT-PCR of ZIKV-infected retinal endothelial cells 96 h after infection. Mock-infected controls are shown, and all values were normalized to GAPDH

**Fig. 4:**
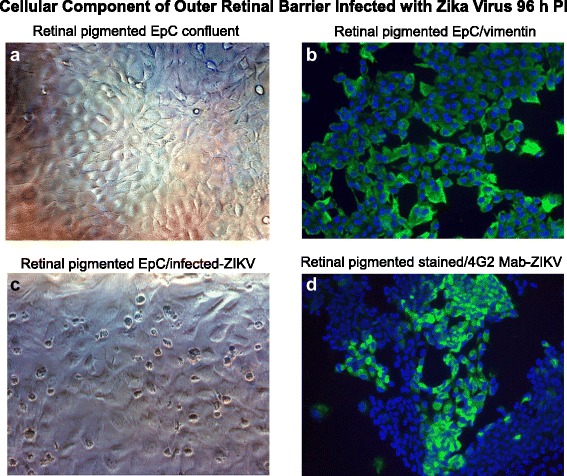
Retinal pigmented epithelial cells and ZIKV infectivity. Phase contrast images of **a** mock-infected confluent monolayer of retinal endothelia cells. **b** Immunofluorescence staining of normal retinal pigmented epithelial with an antibody to vimentin. **c** Phase contrast image of retinal pigmented epithelial cells infected with ZIKV 96 h after infection. **d** Immunofluorescence staining of ZIKV-infected retinal pigmented epithelial cells with the 4G2 antibody. All images were taken on a Nikon TE2000S microscope mounted with a charge-coupled device (CCD) camera at ×200 magnification. For fluorescent images, 4′,6-diamidino-2-phenylindole (DAPI) was used to stain the nuclei blue

**Fig. 5:**
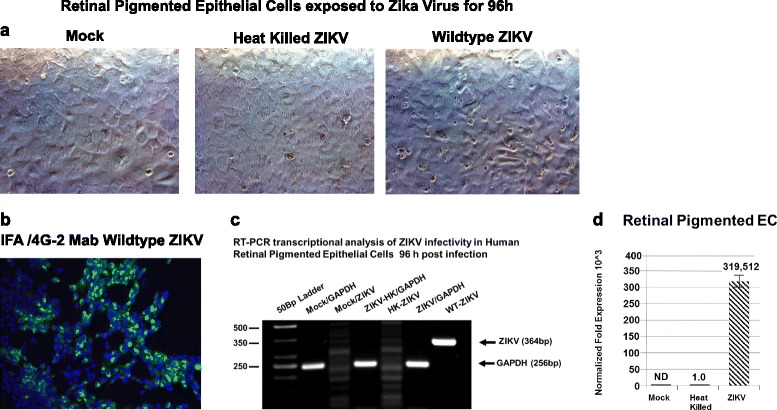
Retinal pigmented epithelial cells infectivity for ZIKV confirmed by RT-PCR. Phase contrast images of: **a** mock-infected confluent monolayer of retinal pigmented epithelial cells, a confluent monolayer of retinal pigmented epithelial cells exposed to heat-killed ZIKV, and retinal pigmented epithelial cells exposed to wild-type ZIKV. **b** Immunofluorescence staining of ZIKV-infected retinal pigmented epithelial cells with the Flavivirus 4G2 antibody. **c** Semiquantitative RT-PCR amplification of a 364-bp fragment using ZIKV-specific primers. GAPDH was amplified as a control represented as a 256-bp fragment. Phase and fluorescent images were taken on a Nikon TE2000S microscope mounted with a charge-coupled device (CCD) camera at ×200 magnification. For fluorescent images, 4′,6-diamidino-2-phenylindole (DAPI) was used to stain the nuclei blue. **d** qRT-PCR of ZIKV-infected retinal pigmented epithelial cells 96 h after infection. Mock-infected controls are shown, and all values were normalized to GAPDH

**Fig. 6:**
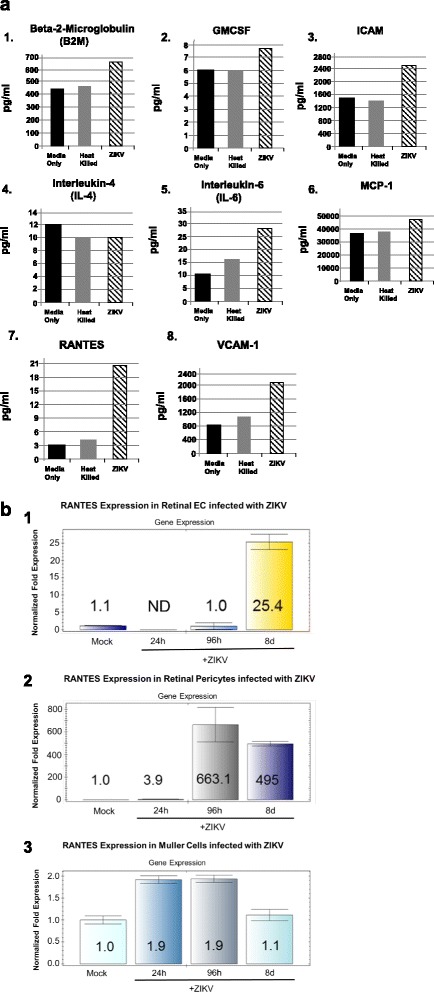
**a** ZIKV induction of proinflammatory and angiogenic cytokines in retinal microvascular endothelial cells after 96 h. Cytokine profiles of ZIKV-infected retinal endothelial cells by Luminex analysis at 96 h post infection are given. Results from cells exposed to media only are shown as *solid black bars*; cells exposed to heat-killed ZIKV are shown as *gray bars*; and results from cells exposed to the ZIKV are shown as *stippled black bars*. Results are included for β2-m (**a-1**), GMCSF (**a-2**), ICAM-1 (**a-3**), IL-4 (**a-4**), and IL-6 (**a-5**), MCP-1 (**a-6**), RANTES (**a-7**), and VCAM-1 (**a-8**). Results are given in picograms per milliliter. The results shown are the averages of replicate samples. **b** RANTES expression levels by qRT-PCR in retinal endothelial cells, retinal pericytes, and Müller cells infected with ZIKV over a time course of 24 and 96 h and 8 days after infection. Results are included for RANTES levels in retinal endothelial cells (**b-1**), RANTES levels in retinal pericytes (**b-2**), and RANTES levels in Müller cells (**b-3**). *Colored bars* indicate the normalized fold transcriptional expression of RANTES compared with mock-infected control cells. *ND* indicates no transcriptional expression detected

**Fig. 7:**
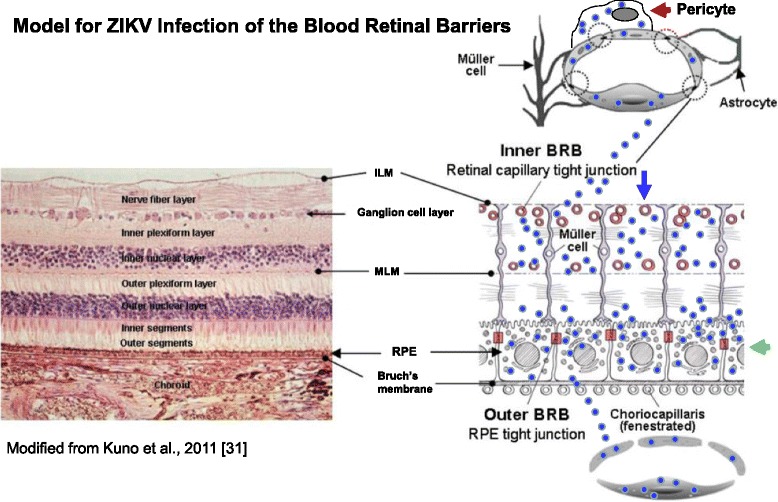
ZIKV blood-retinal barrier infection model. A hypothetical model of ZIKV infection and dissemination from inner and outer blood-retinal barriers into the retinal bed. ZIKV is shown as *blue dots*. ZIKV infects and disseminates from the retinal endothelial cells and retinal pericytes of the IBRB and the retinal pigmented epithelial cells of the OBRB. The *blue arrow* indicates the retinal endothelial cells; the *red arrow* shows the retinal pericytes in the IBRB; and the retinal pigmented epithelial cells in the OBRB are shown by the *green arrow. ILM* internal limiting membrane, *MLM* middle limiting membrane, *RPE* retinal pigmented epithelial cells

We then compared endothelial cell infectivity for ZIKV with retinal pericytes and Müller cells (Fig. 2a-1–a-12). Retinal endothelial cells were found to be highly permissive for ZIKV (Fig. 2a-1–a-3) and showed cytopathic effects that included cells lysis, rounding and sloughing of cells with a more cytoplasmic staining profile when compared with ZIKV-infected retinal pericytes (Fig. 2a-5–a-7). Müller cells (Fig. 2a-9–a-11) were shown not to be permissive for ZIKV infection and did not exhibit ZIKV cytopathic effects 96 h after infection and stained negative with the 4G2 antibody (Fig. 2a-8, 2a-9). Mock-infected controls of retinal endothelial cells, retinal pericytes, and Müller cells stained with the 4G2 antibody are shown in Fig. 2a-4, a-8, a-12, respectively. We then examined ZIKV messenger RNA (mRNA) expression by qRT-PCR over a time course of 24 and 96 h and 8 days after in retinal endothelial cells, retinal pericytes, and Müller cells (Fig. 2b). We observed the highest level of ZIKV mRNA expression by qRT-PCR in pericytes compared to that in retinal endothelial cells, and no ZIKV mRNA expression in Müller cells (Fig. 2b). Normalized fold expression designated as ND refers to ZIKV transcripts not detected via amplification. In addition, the normal fold expression levels for Müller cells exposed to ZIKV in 24 and 96 h are a result of low CT values below threshold levels of reliable amplification.

To further confirm viral infectivity, we examined mock-infected retinal endothelial cells, retinal endothelial cells exposed to heat-killed ZIKV, and retinal endothelial cells exposed to wild-type ZIKV for 96 h (Fig. 3a). We show positive staining for the 4G2 antibody with ZIKV wild-type only (Fig. 3b). Virus-infected retinal endothelial cells showed perinuclear staining with the Flavivirus 4G2 antibody (Fig. 3b). ZIKV infection of retinal endothelial cells was confirmed by RT-PCR using ZIKV-specific oligonucleotide primers (Fig. 3c). We showed semiquantitative RT-PCR amplification of a 364-bp DNA fragment using ZIKV-specific primers, and no amplification using cDNA from total RNA obtained from retinal endothelial cells mock-infected or retinal endothelial cells exposed to heat-killed ZIKV (Fig. 3c). GAPDH was amplified as a control represented as a 256-bp DNA fragment (Fig. 3c). We then examined retinal endothelial cells and controls by qRT-PCR. Our semiquantitative RT-PCR data that showed specific amplification of ZIKV transcripts in ZIKV-infected retinal endothelial cells was validated by qRT-PCR that showed a 13,187-fold increase in ZIKV mRNA amplification compared to mock-infected cells and a 3878-fold increase when compared to heat-killed virus controls (Fig. 3d).

### Retinal pigmented epithelial cells of the OBRB are permissive for ZIKV infectivity and exhibit low-level cytopathology

The structural integrity of the OBRB is established by the tight junctions maintained between retinal pigmented epithelial cells that are proximal to the choroid capillaries which represent another gateway to the retina. We exposed primary human retinal pigmented epithelial cells to ZIKV at a MOI of 0.1 for 96 h. In mock-infected cells, we observed the normal morphology of the retinal pigmented cells in confluent monolayer cultures (Fig. 4a). Retinal pigmented cells stained positive for vimentin as suggested by the supplier (Fig. 4b). Retinal pigmented cells exposed to ZIKV in 96 h produced characteristic ZIKV cytopathic effects (Fig. 4c). ZIKA infectivity was confirmed by immunofluorescent staining using the 4G2 monoclonal antibody after 96 h (Fig. 4d). Virus-infected retinal pigmented epithelial cells showed a perinuclear staining profile with the 4G2 antibody (Fig. 4d).

To further confirm viral infectivity, we examined mock-infected retinal pigmented epithelial cells, retinal pigmented epithelial cells exposed to heat-killed ZIKV, and retinal pigmented epithelial cells exposed to wild-type ZIKV for 96 h (Fig. 5a). We found positive staining for the 4G2 antibody with ZIKV wild-type only (Fig. 5b). Virus-infected retinal pigmented epithelial cells showed perinuclear staining with the Flavivirus 4G2 antibody (Fig. 5b). ZIKV infection of retinal endothelial cells was confirmed by RT-PCR using ZIKV-specific oligonucleotide primers (Fig. 5c). We show semiquantitative RT-PCR amplification of a 364-bp DNA fragment using ZIKV-specific primers, and no amplification using cDNA from total RNA obtained from retinal pigmented epithelial cells mock-infected or retinal pigmented epithelial cells exposed to heat-killed ZIKV (Fig. 5c). GAPDH was amplified as a control represented as a 256-bp DNA fragment (Fig. 5c). We also confirmed our semiquantitative RT-PCR data by qRT-PCR. We observed a 319,512-fold increase in ZIKV mRNA amplification in retinal pigmented epithelial cells compared to controls (Fig. 5d). All values were normalized to GAPDH.

### Dysregulation of angiogenic and proinflammatory cytokines in ZIKV-infected retinal endothelial cells

Angiogenic and proinflammatory cytokine and adhesion molecule levels were examined in retinal endothelial cells exposed to ZIKV for 96 h (Fig. 6a). In retinal endothelial cells exposed to ZIKV, we observed only a marginal increase in levels of β2-m, GMCSF, and MCP1; a moderate increase of ICAM-1, IL-6, and VCAM-1 expression; but a strong increase of RANTES expression when to mock-infected controls (Fig. 6a-1–a-8). We observed moderately higher levels of IL-4 in mock-infected cells compared to that in ZIKV-infected retinal endothelial cells (Fig. 6a-4). In retinal endothelial cells exposed to heat-killed virus, we observed lower levels of β2-m, GMCSF, ICAM-1, IL-6, MCP1, RANTES, and VCAM-1 compared to ZIKV-exposed cells (Fig. 6a-1–a-3, a-5–a-8). Following the significant increase in RANTES expression levels in retinal endothelial cells by Luminex analysis, we performed qRT-PCR for RANTES transcription levels in ZIKV-infected retinal endothelial cells, retinal pericytes, and Müller cells over a time course of 24 and 96 h and 8 days after ZIKV infection (Fig. 6b). In retinal endothelial cells, we observed the highest level (25-fold) of RANTES expression in ZIKV-infected cells in 8 days when compared to that in mock-infected cells (Fig. 6b-1). No significant RANTES transcriptional expression was observed in 24 and 96 h when compared to that in mocked-infected cells (Fig. 6b-1). In retinal pericytes, we observed the highest level (663-fold) of RANTES transcriptional expression in ZIKV-infected cells in 96 h after infection (Fig. 6b-2). A fourfold increase in RANTES expression was observed in 24 h when compared to that in mock-infected cells, but a reduction in RANTES expression (495-fold) was observed in 8 days when compared with 96 h in ZIKV-infected cells (Fig. 6b-2). We observed only a marginal increase in RANTES transcriptional expression in Müller cells in 24 and 96 h after infection when compared to ZIKV-infected cells after 8 days and mock-infected cells (Fig. 6b-3).

### ZIKV blood-retinal barrier infection model

In this hypothetical model, ZIKV is shown as blue dots (Fig. 7). Based on our initial findings, we have developed a ZIKV blood-retinal barrier infection model [31]. In the model, we propose that ZIKV enters the IBRB via the retinal arteries and subsequently the retinal capillaries (Fig. 7). There is infection and virus amplification in retinal endothelial cells (blue arrow) of the retinal capillaries and retinal pericytes (red arrow) that are abluminal to retinal endothelial cells allowing the virus to enter the inner retinal bed. We also propose in the model that ZIKV enters the OBRB via the choroid capillaries (Fig. 7). The choroid is a highly vascularized tissue that supplies blood to the retina, and the choroidal capillary endothelial cells in humans are highly fenestrated which would allow ZIKV ready access to permissive retinal pigmented epithelial cells [31] (Fig. 7). Infection and virus amplification in retinal pigmented epithelial cells (green arrow) ensue allowing viral dissemination into the proximal retinal bed (Fig. 7).

## Discussion

There is no information in the literature that defines target cell populations in the human eye related to ZIKV-associated ocular disease. This study provides information that is important for understanding ZIKV pathology in the ocular compartment and identifies important cell types in both the inner and outer blood-retinal barriers (IBRB and OBRB) that are permissive for ZIKV infection and dissemination in the eye. This in vitro study suggests that ZIKV traffics both retinal endothelial cells, retinal pericytes, and retinal pigmented epithelial cells during infection but does not infect Müller cells. The highest levels of ZIKV transcription were observed in retinal pericytes compared in retinal pigmented epithelial cells and retinal endothelial cells. The model we proposed is hypothetical because primary cells in culture may not behave as cells in ocular tissue and will require validation in vivo. Cytokine and adhesion molecule profile analysis reveals a marginal increase in the levels of β2-m, GMCSF, and MCP1 and a moderate increase of ICAM-1, IL-6, and VCAM-1 expression; however, significantly higher levels of RANTES expression were observed in ZIKV-infected cells compared in controls (Fig. 6a). Recent studies show that patients with ZIKV infection have high levels of RANTES in their serum when compared with patients infected with dengue virus or Chikungunya virus [32]. Upregulation of RANTES over time would lead to chronic inflammation and recruitment of inflammatory cells in the retinal microenvironment. The next steps for this study will be to directly examine eye washing or lacrimal fluid from patients with ZIKV-associated ocular hyperemia or ocular tissue from infants who have died of congenital ZIKV infection to determine viral dissemination patterns and cytokine expression profiles in vivo.

## Conclusions

We have identified primary human retinal endothelial cells and retinal pericytes of the IBRB and human retinal pigmented epithelial cells of the OBRB as target cells for ZIKV infection in the eye. We have determined that ZIKV induces a moderate angiogenic and proinflammatory cytokine response with the exception of RANTES in infected retinal endothelial cells that likely play a major role in ocular inflammation in acute ZIKV ocular disease. The hypothetical model we proposed based on our findings suggests that ZIKV disseminates throughout the retinal bed via the retinal arteries and infects retinal capillary endothelial cells and retinal pericytes of the IBRB and traffics the choroid capillaries to infect retinal pigmented epithelial cells in the OBRB.

## Abbreviations

4G2 antibody: Flavivirus group antigen monoclonal antibody
β2-m: Beta-2 microglobulin
BBB: Blood brain barrier
bp: Base pair
CCD: Charge-coupled device camera
CDC: Centers for Disease Control and Prevention
cDNA: Complementary DNA
DAPI: 4′,6-diamidino-2-phenylindole
DMEM: Dulbecco’s Modified Eagle Medium
EpiCM: Epithelial cell medium
FITC: Fluorescein isothiocyanate
GAPDH: Glyceraldehyde 3-phosphate dehydrogenase
GMCSF: Granulocyte macrophage colony-stimulating factor
IBRB: Inner blood-retinal barrier
ICAM-1: Intercellular adhesion molecule 1
IL-4: Interleukin-4
IL-6: Interleukin-6
MCP-1: Monocyte chemotactic protein-1
MIO-M1: Müller cell line from human retina
MOI: Multiplicity of infection
OBRB: Outer blood-retinal barrier
PBS: Phosphate-buffered saline
PCR: Polymerase chain reaction
Pen/Strep: Penicillin streptomycin mixtures
PRVABC59: Asian strain of Zika virus isolated in Puerto Rico in December 2015 from human serum
qRT-PCR: Quantitative reverse transcription polymerase chain reaction
RANTES: Regulated upon activation normal T cell expressed and presumably secreted
RT-PCR: Reverse transcription polymerase chain reaction
VCAM-1: Vascular cell adhesion molecule 1
VWF: von Willebrand factor
ZIKV: Zika virus
